# Robust high-Q filter with complete transmission by conjugated topological photonic crystals

**DOI:** 10.1038/s41598-020-64076-3

**Published:** 2020-04-27

**Authors:** Yu-Chuan Lin, Shih-Han Chou, Wen-Jeng Hsueh

**Affiliations:** 1 Photonics Group, Department of Engineering Science and Ocean Engineering, National Taiwan University, 1, Sec. 4, Roosevelt Road, Taipei, 10660 Taiwan; 2 Taiwan Instrument Research Institute, National Applied Research Laboratories, 20, R&D Rd. VI, Hsinchu Science Park, Hsinchu, 30076 Taiwan

**Keywords:** Optics and photonics, Applied optics, Optical physics, Optical techniques

## Abstract

High quality factor (High-Q) and transmission optical devices are required for various applications in the fields of physics and engineering. Critical for these applications is the realization of a structure with high-Q, complete transmission and small volume. A robust high-Q filter with complete transmission by conjugated topological photonic crystals (CTPC) is presented. The study shows that an ultra-high-Q of more than 10^8^ with complete transmission is obtained by the CTPC with 2 μm long due to the excitation of conjugated topological edge-states (CTES). It is also found that even though the quality factor of resonances increases as the periodic number of multilayers increases, these resonances are still complete transmission. A novel concept of CTES is first proposed in this study and investigated the effect of its topological phenomenon on high quality factor via CTPC. We theoretically realize the robust high-Q and complete transmission in the CTPC, which is different from those in periodic, quasi-periodic, Fabry-Perot photonic crystals and traditional topological photonic crystals (TPC).

## Introduction

High-Q and complete transmission has attracted more attention since it is very promising for applications in advanced narrowband selective filters, optical switches, modulators, sensors, communications and quantum information processing^1–5^. High-Q leads to superior spectral resolution and improved sensitivity. Nevertheless, high-Q will typically result in attenuation of the transmission due to both the number of reflections inside the device and the increases in the light path^6,7^. This phenomenon greatly limits the potential for practical applications. High transmission is of special importance for those applications because the transmission of light can tunnel through the tunneling barrier, without loss of power, but light with other frequencies is reflected. However, in traditional photonic crystals (PCs) and waveguides, a high-Q transmission is accompanied by an increased sensitivity to manufacturing imperfections, in order that light is more likely scattered and localized by disorder, especially, for example, Fabry-Perot resonators and micro-cavities based on Bragg mirrors photonic crystals^8–10^, which causes the limited potential for practical application.

Recently, there has been a surge in attempts to implement topological protection in photonic crystals, due to the application potential of robust transmission, resist backscattering, against perturbations and strong field localization^11–14^. Topological edge-states (TES) exist at the interface of the hetero-structure between two photonic crystals, which, simulating quantum spin Hall effects, have been shown to robust edge conductance and topological protection^15,16^. This phenomenon is one of the interesting topics in recent years, because it brings new theoretical discoveries and more abundant potential applications^17–21^. Nevertheless, there are very few suggestions for technical applications of topological phenomenon in high-Q filters. Studies on the use of topological edge-states to generate robust high-Q and complete transmission by use of all-dielectric photonic crystals are less discussed. In the past few years, Fabry–Perot cavity and a cavity made of Bragg mirrors are common types used to generate high-Q. In previous reports, a quality factor of 10^6^ was achieved by use of Fabry-Perot cavity, but the transmission decreases to the range between 0.2 and 0.7^6,7^. A quality factor exceeding of 10^7^ was realized by the photonic crystal nano-cavities^22^ and the topological Fano resonance^23^, but the transmission was reduced to about 0.8. Unfortunately, an integration scheme that achieves both high-Q and high transmission has not yet been shown. Therefore, a sustained effort to enhanced quality factor and maintain transmission is the main task for realizing excellent optical performance.

In this article, we attempt to overcome the problem of transmission decreases caused by quality factor increases. The study first presents a novel concept of the CTES to achieve high-Q transmission by the CTPC. A robust high-Q filter with complete transmission is theoretically realized. The problem of transmission decreases result from quality factor increases in traditional photonic crystals is significantly improved by this study. Furthermore, the correlation between the quality factor, the transmission spectra, the period number of multilayers and the thickness variations is discussed. We found that even though the quality factor of resonances increases as the periodic number of multilayers increases, these resonances are still perfect transmission. The superior quality factor and transmission are obtained as the parameters of TPC are conjugated. These resonance peaks have robust and complete transmission exactly equal to 1 characteristic, which is different from those in traditional TPC. Although various studies on one-dimensional photonic crystals have been popular in recent years, research on topological protection in one-dimensional photonic crystals has only recently emerged. There are very few suggestions for scientific applications of topological phenomena in optical filters. Studies on the use of topological interface states to obtain robust high transmission by use of all-dielectric topological photonic crystals have hardly been discussed. It is worth mentioning that the concept of CTPC was first proposed in this study and investigated the effect of its topological phenomenon on high quality factor. We theoretically realize the high-Q and complete transmission in CTPC. Furthermore, the problem of transmission decreases result from quality factor increases in traditional photonic crystals is significantly improved by this study. It is very useful to develop advanced a high-Q and perfect transmission filter.

## Model and Method

**Figure 1:**
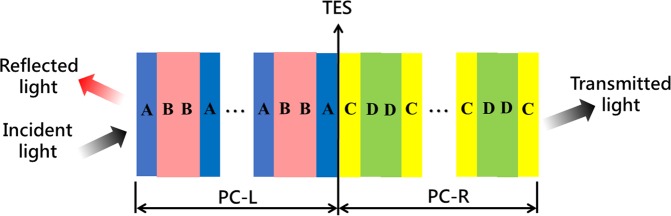
Schematic diagram of the one-dimensional CTPC consists of PC-L and PC-R.

**Figure 2:**
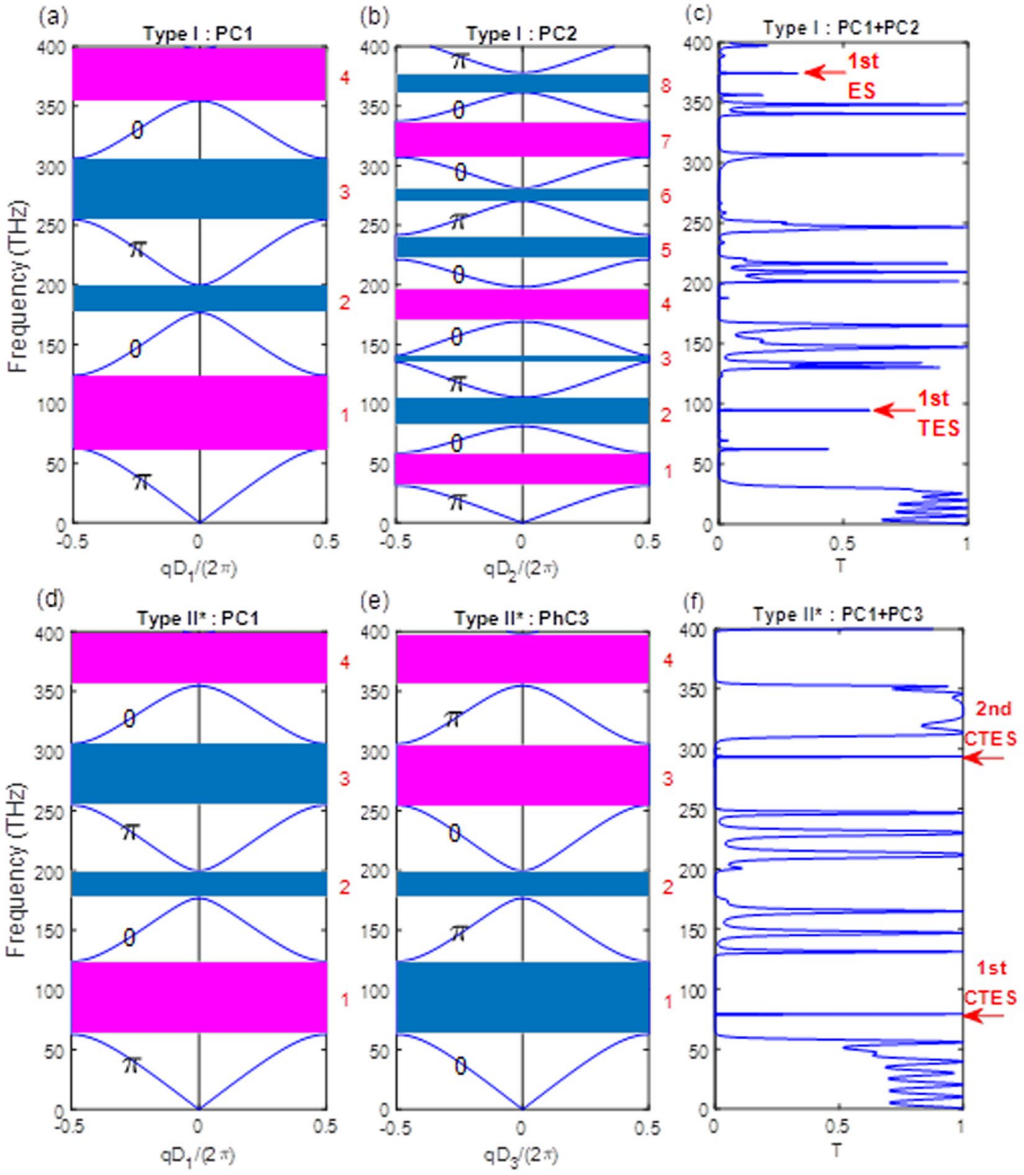
(**a**) Type I: Band structure and Zak phases of PC 1, (**b**) Type I: Band structure and Zak phases of PC 2 **(c)** Type I: transmission spectrum of PC 1 and PC 2. (**d**) Type II*: Band structure and Zak phases of PC 1, (**e**) Type II*: Band structure and Zak phases of PC 3 **(f)** Type II*: transmission spectrum of PC 1 and PC 3. Type I: the parameters of the considered structure are n_A_ = 3, n_B_ = 1, n_C_ = 3, n_D_ = 1, n_i_ = n_o_ = 1, F1 = 0.3, F2 = 0.38. Type II*: the parameters of considered structure are n_A_ = 3, n_B_ = 1, n_C_ = 1, n_D_ = 3, n_i_ = n_o_ =1, F1 = 0.3, F3 = 0.7.

Initially, we consider one-dimensional topological photonic crystals composed of alternatively placed high and low dielectric constant materials. As shown in Fig. 1, the one-dimensional topological photonic crystals consist of PC-L and PC-R. The constituent photonic crystals PC-L has a configuration of (ABBA)^N^. The constituent photonic crystals PC-R has a configuration of (CDDC)^N^. In order to verify the robust high-Q and perfect transmission caused by the excitation of topological edge-states, we show a relation between the reflection phases and Zak phases of one-dimensional topological photonic crystals. These can be used to determine the existence of topological edge-states at the interface of the two photonic crystals, PC-L and PC-R in a particular band gap. The topological phase can be represented by the following equation^24–26^.

1 $${\theta }_{n}^{Zak}={\int }_{-\pi /\varLambda }^{\pi /\varLambda }[i\mathop{\int }\limits_{unit\,cell}dz{\epsilon }(z){u}_{n,q}^{\ast }(z){\partial }_{q}{u}_{n,q}(z)]dq$$

where ε (z) denotes the dielectric function, and u_n,q_(z) represents the Bloch eigen-function of electric field at the n-th photonic band gap with a Bloch wave-vector q. One-dimensional topological photonic crystals with inversion symmetry always have the Zak phase quantized at either 0 or π. We determine that the TES can be excited when the phase changes from 0 to π or from π to 0. In order to verify that the topological edge-states exist at the interface of the one-dimensional topological photonic crystals, our study shows the band structure, Zak phase and transmission spectrum as in Fig. 2.

It is assumed that all of the layers and bonding media in the structure are uniform and optically isotropic without absorption. The thicknesses of layers A, B, C and D are denoted as d_A_, d_B_, d_C_, and d_D_, respectively. The thickness of the system is denoted as d, where d1 = d_A_ + d_B_ and d2 = d_C_ + d_D_. The normalized frequency is denoted as Ω = ωD/2πc, where c is the speed of light in a vacuum. The periodic number and thickness filling factor of the structure are denoted as N and F, respectively, where F1 = d_A_/d1 and F2 = d_C_/d2. The indices of the dielectric layers A, B, C and D are denoted, respectively, as n_A_, n_B_, n_C,_ and n_D._ The indices of semi-infinite bonding media are n_i_ and n_O_ respectively. Moreover, we classified the parameters of the considered structure into two types. Type I is n_A_ = 3, n_B_ = 1, n_C_ = 3, n_D_ = 1, n_i_ = n_o_ =1, F1 = 0.3, F2 = 0.38, d1 = 1 μm, d2 = 1.85 μm. Type II* is n_A_ = 3, n_B_ = 1, n_C_ = 1, n_D_ = 3, n_i_ = n_o_ =1, F1 = 0.3, F3 = 0.7, d1 = d3 = 1 μm. The theoretical model and method is developed to explain a topological phenomenon in the CTPC and further, to be able to forecast it. The refractive index of the dielectric layers B and D in this study model is assumed to be 1, which is mainly a consideration of theoretical calculation and a clear description. These assumptions can represent the theoretical model and parameter settings of this study, but they will not affect the correctness of the results.

## Results and Discussions

From the study results, the topological edge-states can be verified by the band structure of two PCs and obtained in the transmission spectrum. Figure 2(a,b) show the band structure of Type I for PC1 and PC2. The red band represents a band gap with a positive topological phase, while the blue implies a band gap with negative topological phase. In addition, the Zak phase of each individual band gap is labeled at the center of its own band. It can be seen that the gaps of PC1 and PC2 have different signs of topological properties. A topological edge-state exists at the interface if the sign is opposite. This represents a topological phase transition, which occurs when two bands cross each other. We can also see that the topological edge-state has been excited when the Zak phase changes from 0 to π or from π to 0. Figure 2(c) clearly shows the resonance transmission spectrum of topological photonic crystals. The transmission peaks are obtained at 98 THz, as shown by the red arrow pointing at the 1st TES. In addition, we also found that the edge-state (ES) appears in this Type I as indicated by the red arrow pointing at the 1st ES. The main difference between TES and ES is the Zak phase change. TES must meet the reflection and Zak phase change. ES only meets the reflection change, without meeting the Zak phase change. Figure 2(d,e) show the band structure of Type II* for PC1 and PC3. We can see the reflection phase from the supplementary information. We observe that there are two CTES generated in the band gap of 1 and 3. It is clear from Fig. 2(f) that the two CTES are excited at 75 THz and 290 THz. Moreover, a high-Q of 10^8^ with perfect transmission is obtained by the TPC due to the excitation of CTES. The correlation between the Q factor, the transmission, the periodic number of multilayers, and the thickness variations is discussed based on the two types.

**Figure 3:**
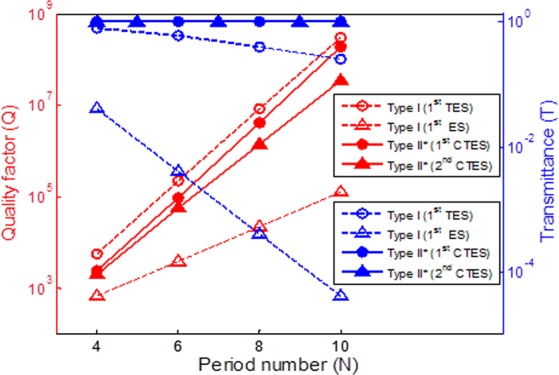
The maximum value of quality factor and the transmittance versus the period number variation for topological photonic crystals with different types. All of the parameters are the same as those used in Fig. 2. Type I is non-conjugated with F1 = 0.3 and F2 = 0.38. Type II* is conjugated with F1 = 0.3 and F2 = 0.7.

To study optical transmission characteristics of topological photonic crystals, we show the quality factor versus the periodic number variation for different types and edge-states. In Fig. 3, the red dashed curves with circle and triangle marks show the relationship between quality factor and periodic number variation for Type I 1^st^ TES and 1^st^ ES. The red solid curves with circle and triangle marks show the relationship between quality factor and periodic number variation for Type II* 1^st^ CTES and 2^nd^ CTES. Similarly, the blue solid and dashed curves represent transmittance. It can be seen that the quality factor increases as the periodic number increases. Moreover, we found that the 1^st^ TES and 1^st^ CTES have better quality factor in the two types. It is also found that the quality factor of Type I is superior to Type II*, but the transmittance of Type I is not as good as that of Type II*. The transmittance of Type I decrease as the periodic number of multilayers increases, while the transmittance of Type II* still maintain perfect transmittance exactly equal to 1. Type II* also has superior transmittance than that in previous reports [6–7] as the quality factor increases.

**Figure 4:**
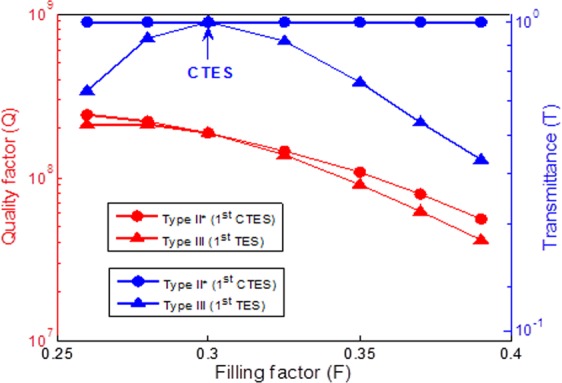
The maximum value of quality factor and the transmittance versus the thickness filling factor variation for topological photonic crystals with different types. Type II* is conjugated, the parameters of the considered structure are n_A_ = 3, n_B_ = 1, n_C_ = 1, n_D_ = 3, n_i_ = n_o_ =1, F1 from 0.26 to 0.39, F3 is conjugated with F1 variation. Type III is non-conjugated, the parameters of considered structure are n_A_ = 3, n_B_ = 1, n_C_ = 1, n_D_ = 3, n_i_ = n_o_ =1, F1 from 0.26 to 0.39, F2 is fixed at 0.7.

Next, we turn our attention to the effect of the thickness filling factor for two PCs with different types. The parameters of Type II* in Fig. 3 are adopted and subdivided into two types. Type II* is conjugated, and the parameters of considered structure are n_A_ = 3, n_B_ = 1, n_C_ = 1, n_D_ = 3, n_i_ = n_o_ =1, F1 from 0.26 to 0.39, and F3 is always conjugated with F1 variation. Type III is non-conjugated, and the parameters of the considered structure are n_A_ = 3, n_B_ = 1, n_C_ = 1, n_D_ = 3, n_i_ = n_o_ =1, F1 from 0.26 to 0.39, and F3 is fixed at 0.7. The quality factor and transmittance versus different thickness filling factors for two types of topological photonic crystals is shown in Fig. 4. The red solid curves with circle and triangle marks show the relationship between quality factor and thickness filling factor variation for Type II* and Type III, respectively. Similarly, the blue solid curves with circle and triangle marks represent transmittance. We can observe that the thickness filling factor have significant effect on the quality factor. The quality factor increases as the thickness filling factor decreases. It was also found that in Type II* has superior quality factor at the same thickness filling factor. The maximum value of the quality factor of 2.25 × 10^8^ is achieved for the Type II* of F1 = 0.26 and F2 = 0.74, and F3 is conjugated with F1 variation. In addition, the transmittance of Type II* does not change with thickness filling factor variation and maintains perfect transmission exactly equal to 1. However, the transmittance of Type III is greatly affected by thickness filling factor variation. Type III has the maximum value of the transmittance, as indicated by the blue arrow, but it decreases rapidly as the thickness filling factor changes to non-conjugated.

**Figure 5:**
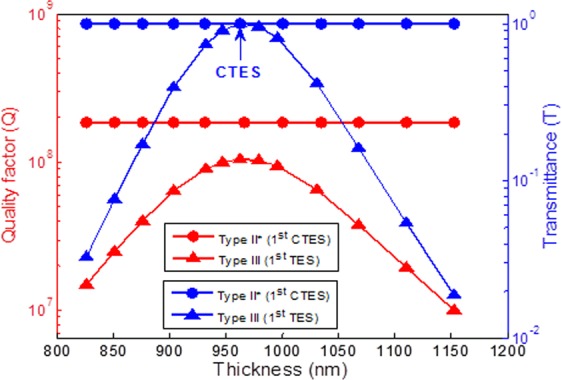
The maximum value of quality factor and the transmittance versus the thickness of PhC1 variation for topological photonic crystals with different types. All of the parameters are the same as those used in Fig. 4. Type II* is non-conjugated, F1 = 0.3, and F3 = 0.7. Type III is non-conjugated, F1 = 0.35, and F3 = 0.7.

The quality factor and the transmittance versus the thickness of PC1 variation for two PCs with different types are investigated as shown in Fig. 5. All of the parameters are the same as those used in Fig. 4. Type II* is conjugated, F1 is 0.3, and F3 is 0.7 conjugated with F1. Type III is non-conjugated, F1 is 0.35, and F3 is 0.7. We found that the thickness of PC1 variation has a significant effect on the quality factor and transmittance in Type III. The curve of quality factor and transmittance versus thickness of PC1 variation shows an inverted U-like shape. The maximum value of quality factor of 1.05 × 10^8^ at 962.60 nm is obtained. Meanwhile, the transmittance of 1 is reached at the same thickness, as indicated by the blue arrow. However, the thickness of PC1 variations has a minor effect on the quality factor and transmittance for Type II*. The maximum value of quality factor of 1.86 × 10^8^ and perfect transmission exactly equal to 1 is obtained at any thickness. A brief conclusion is drawn that the robust quality factor and high transmittance are obtained as the parameters of topological photonic crystals are conjugated. Based on our design and relevant numerical simulations in this article, we can fabricate filter devices by magnetron sputtering and photolithography technique. The possible experimental strategy to demonstrate this high-Q complete transmission is that the measurement of transmission spectrum can carry out by sweeping the wavelength of a tunable diode laser and recording the transmitted power. In addition, it can be expected that the Q factor will be sharply decreased in the experiment if the structure is not conjugated topological photonic crystals.

**Figure 6:**
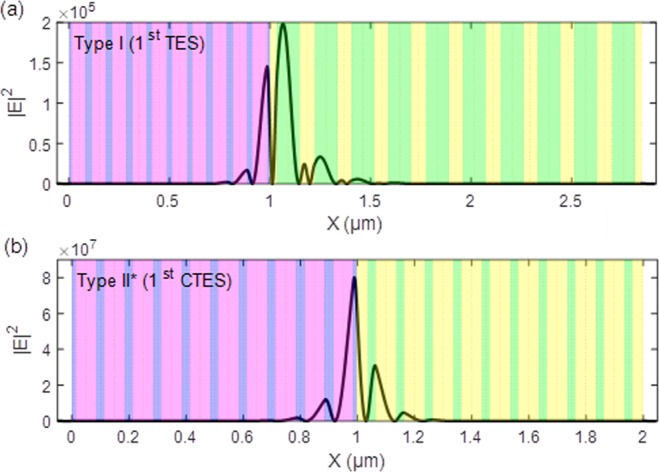
lEl^2^ distribution at complete transmission resonances in 1st TES and 1st CTES. (**a**) lEl^2^ distribution at Type I. (**b**) lEl^2^ distribution at Type II*. The blue regions represent dielectric material A and the red regions represent dielectric material B, the green regions represent dielectric material C and the yellow regions represent dielectric material D, the white regions represent air. All of the parameters are the same as those used in Fig. 2, and the periodic numbers N = 10.

The squared electric field distribution of the 1^st^ TES and 1^st^ CTES resonance modes at Type I and Type II* are illustrated in Fig. 6. The blue regions represent dielectric material A and the red regions represent dielectric material B; the green regions represent dielectric material C and the yellow regions represent dielectric material D; the white regions represent air. All of the parameters are the same as those used in Fig. 2. The electromagnetic wave propagates from the left-hand side to the right-hand side for normal incidence. The symbol X on the x-axis denotes the total length of topological photonic crystals. It can be seen that the electric field is evanescent and passes through topological photonic crystals at the interface of the two PCs. Moreover, we found that the light is strongly localized in the interface between the two photonic crystals due to the excitation of topological edge-states. There is a high squared electric field distribution of the 1^st^ CTES resonance modes in Type II*. The maximum squared electric field intensity of Type II* is 8 ×10^7^, which is greater than that in Type I. Topological edge-states exist at the interface between two photonic crystals, which is different from traditional Fabry-Perot resonators and micro-cavities based on Bragg mirrors photonic crystals. TES of topological photonic crystals simulating the quantum spin Hall effects have been shown to produce robust edge conductance and topological protection rather than a standing wave. The electric field distribution of TES shows an asymmetric shape, which is also different from traditional Fabry-Perot resonators. The basic principle of preserve complete transmission in high-Q system is that the CTPC structure can significantly enhance resonance or localization state at interface of the CTES. The reason for the enhanced resonance or localization state is that as the light from the left side reaches the photonic structures, part of it is reflected back to the structure. This reflection is equivalent to enhancing the boundary reflection coefficient of the multi-layers. It is similar to the resonance in a Fabry-Perot resonator, which is based on internal multi-reflection of the light between the two reflectors. Constructive interference occurs if the two beams are in phase, leading to resonant enhancement of light inside the resonator. Therefore, the stronger interface mode resonance enhances the localization state, as well as the transmission. In addition, these CTES resonance modes of topological photonic crystals can be utilized to enhance light-matter interaction and non-linear optics, which improves the light transmission performance. The strong light-matter interaction in integrated high-Q optical devices makes them ideal for communications and quantum information processing.

## Conclusions

In this article, we have proposed a robust high-Q filter with complete transmission by use of one-dimensional conjugated topological photonic crystals. It is of great significance to improving the quality factor and the transmittance in applications for advanced narrowband selective filters. The excitations of topological edge-states in one-dimensional photonic crystals systems are robust against the introduced interfacial perturbations and excited resonance mode, which is different from those in periodic, quasi-periodic, Fabry-Perot photonic crystals and traditional topological photonic crystals. We found that ultra-high-Q of more than 10^8^ with perfect transmission is obtained by one-dimensional CTPC with 2 μm long due to the excitation of CTES. It is worth mentioning that even though the quality factor of resonances increases as the periodic number of multilayers increases, these resonances are still of perfect transmission. Moreover, a robust ultra-high-Q and perfect transmittance is obtained as the parameters of topological photonic crystals are conjugated. These resonance peaks have robust and complete transmission exactly equal to 1 characteristic. This special advantage makes the CTPC desirable in advanced ultra-high-Q filters, optical switches, modulators, sensors, communications and quantum information processing.

## Supplementary information

Supplementary information.
