# Supplementary material for: Robust high-Q filter with complete transmission by conjugated topological photonic crystals
Source: Sci Rep. 2020 Apr 27;10:7040. doi: 10.1038/s41598-020-64076-3 (PMC7184720; doi:10.1038/s41598-020-64076-3)
Supplement: Supplementary file 1 — Supplementary information. [file 41598_2020_64076_MOESM1_ESM.docx]

**Robust high-Q filter with complete transmission by conjugated topological photonic crystals**

# Yu-Chuan Lin1,2, Shih-Han Chou^1^, and Wen-Jeng Hsueh1*

1 Photonics Group, Department of Engineering Science, National Taiwan University, 1, Sec. 4, Roosevelt Road, Taipei, 10660, Taiwan

2 Taiwan Instrument Research Institute, National Applied Research Laboratories, 20, R&D Rd. VI, Hsinchu Science Park, Hsinchu, 30076, Taiwan

* hsuehwj@ntu.edu.tw

**SUPPLEMENTARY**

This document provides supplementary information to high-Q filter with complete transmission by conjugated topological photonic crystals. The topological edge-state is related to the topological properties of two photonic crystals in the band gap. However, the reflection phases and surface impedance directly govern the topological properties. In this supplement, we show the reflection phases of two types of topological photonic crystals. When the reflection phase has the same sign, the Zak phase is π, otherwise it is 0. Fig. 1 (a) and (b) show the reflection phases of type I for PC1 and PC2. The parameters of the considered structure are n_A_ = 3, n_B_ = 1, n_C_ = 3, n_D_ = 1, n_i_ = n_o_ =1, F1 = 0.3, F2 = 0.38.

Fig. 2 (a) and (b) show the reflection phases of type II* for PC1 and PC3. The parameters of considered structure are n_A_ = 3, n_B_ = 1, n_C_ = 1, n_D_ = 3, n_i_ = n_o_ =1, F1 = 0.3, F3 = 0.7. Fig. 3 (a) and (b) show the band structure of type III for PC1 and PC3. The red band represents a band gap with a positive topological phase, while the blue implies a band gap with negative topological phase. In addition, the Zak phase of each individual band gap is labeled at the center of its own band. It can be seen that the gaps of PC1 and PC3 have different signs of the topological properties.

There is a topological edge-states exists at the interface if the sign is opposite. This represents a topological phase transition, which occurs when two bands cross each other. We can also seem that topological edge-state have been excited when the Zak phase changes from 0 to π or from π to 0. Fig. 3 (c) clearly shows the resonance transmission spectrum of topological photonic crystals. The transmission peaks are obtained at 75 THz, such as the red arrow pointing 1^st^ TES. Fig. 4 (a) and (b) show the reflection phases of type I for PhC1 and PhC3. The parameters of the considered structure are n_A_ = 3, n_B_ = 1, n_C_ = 1, n_D_ = 3, n_i_ = n_o_ =1, F1 = 0.35, F3 = 0.7. Fig. 4 (a) and (b) show the reflection phases of type III for PC1 and PC3. The parameters of considered structure are n_A_ = 3, n_B_ = 1, n_C_ = 1, n_D_ = 3, n_i_ = n_o_ =1, F1 = 0.35, F3 = 0.7..

Fig. 1. (a) Type I : reflection phases of PC 1, (b) Type I : reflection phases of PC 2. the parameters of the considered structure are n_A_ = 3, n_B_ = 1, n_C_ = 3, n_D_ = 1, n_i_ = n_o_ = 1, F1 = 0.3, F2 = 0.38.

Fig. 2. (a) Type II : reflection phases of PC 1, (b) Type II : reflection phases of PC 3. The parameters of the considered structure are n_A_ = 3, n_B_ = 1, n_C_ = 1, n_D_ = 3, n_i_ = n_o_ =1, F1 = 0.3, F3 = 0.7.

Fig. 3. (a) Type III : Band structure and Zak phases of PC 1, (b) Type III : Band structure and Zak phases of PC 3 (c) Type III : transmission spectrum of PC1 and PC3. The parameters of the considered structure are n_A_ = 3, n_B_ = 1, n_C_ = 1, n_D_ = 3, n_i_ = n_o_ =1, F1 = 0.35, F3 = 0.7.

Fig. 4. (a) Type III : reflection phases of PC1, (b) Type III : reflection phases of PC3. The parameters of the considered structure are n_A_ = 3, n_B_ = 1, n_C_ = 1, n_D_ = 3, n_i_ = n_o_ =1, F1 = 0.35, F3 = 0.7.
